# Development of SARS-CoV-2 Specific IgG and Virus-Neutralizing Antibodies after Infection with Variants of Concern or Vaccination

**DOI:** 10.3390/vaccines9070700

**Published:** 2021-06-25

**Authors:** Franziska Neumann, Ruben Rose, Janine Römpke, Olaf Grobe, Thomas Lorentz, Helmut Fickenscher, Andi Krumbholz

**Affiliations:** 1 Labor Dr. Krause und Kollegen MVZ GmbH, Steenbeker Weg 23, D-24106 Kiel, Germany; neumann@labor-krause.de (F.N.); grobe@labor-krause.de (O.G.); lorentz@labor-krause.de (T.L.); 2 Institut für Infektionsmedizin, Christian-Albrechts-Universität zu Kiel und Universitätsklinikum Schleswig-Holstein, Campus Kiel, Brunswiker Straße 4, D-24105 Kiel, Germany; rose@infmed.uni-kiel.de (R.R.); fickenscher@infmed.uni-kiel.de (H.F.); 3 Städtisches Krankenhaus Kiel, Chemnitzstraße 33, D-24116 Kiel, Germany; janine.roempke@krankenhaus-kiel.de

**Keywords:** COVID-19, virus variants, vaccination, humoral immune response, IgG, titer, virus-neutralizing antibodies, avidity

## Abstract

The humoral immunity after SARS-CoV-2 infection or vaccination was examined. Convalescent sera after infection with variants of concern (VOCs: B.1.1.7, *n* = 10; B.1.351, *n* = 1) and sera from 100 vaccinees (Pfizer/BioNTech, BNT162b2, *n* = 33; Moderna, mRNA-1273, *n* = 11; AstraZeneca, ChAdOx1 nCoV-19/AZD1222, *n* = 56) were tested for the presence of immunoglobulin G (IgG) directed against the viral spike (S)-protein, its receptor-binding domain (RBD), the nucleoprotein (N) and for virus-neutralizing antibodies (VNA). For the latter, surrogate assays (sVNT) and a Vero-cell based neutralization test (cVNT) were used. Maturity of IgG was determined by measuring the avidity in an immunoblot (IB). Past VOC infection resulted in a broad reactivity of anti-S IgG (100%), anti-RBD IgG (100%), and anti-N IgG (91%), while latter were absent in 99% of vaccinees. Starting approximately two weeks after the first vaccine dose, anti-S IgG (75–100%) and particularly anti-RBD IgG (98–100%) were detectable. After the second dose, their titers increased and were higher than in the convalescents. The sVNT showed evidence of VNA in 91% of convalescents and in 80–100%/100% after first/second vaccine dose, respectively. After the second dose, an increase in VNA titer and IgGs of high avidity were demonstrated by cVNT and IB, respectively. Re-vaccination contributes to a more robust immune response.

## 1. Introduction

The pandemic caused by the severe acute respiratory syndrome coronavirus 2 (SARS-CoV-2) [1] is a great challenge for humanity. To date, 157,897,763 confirmed COVID-19 cases have been reported to the World Health Organization (WHO), including 3,287,082 deaths [2]. Safe, effective, and widely available protective vaccines are essential to contain and end this pandemic [3]. Almost all vaccines aim at the induction of neutralizing antibodies, which are directed against the viral spike (S)-protein and in particular against its receptor-binding domain (RBD) and which thereby prevent the infection of the cell [4]. Furthermore, all vaccines should generate a robust cellular immune response [4,5].

So far, four vaccines have received conditional approval in the European Union [6]. These are, on the one hand, the messenger ribonucleic acid (mRNA) vaccines from Pfizer/BioNTech (BNT162b2) and Moderna (mRNA-1273), in each of which the genetic information for the complete SARS-CoV-2 S-protein has been optimized and liposomally packaged to allow that a stable and highly immunogenic protein is expressed after vaccination [3,4]. On the other hand, there are two approved vector vaccines, which use either a replication-deficient rare human adenovirus 26 (Ad26. COV2; Janssen) or a chimpanzee adenovirus (ChAdOx1 nCoV-19/AZD1222; AstraZeneca, in the following designated as AZD1222) to introduce the genetic information of the S-protein into cells followed by the expression of this SARS-CoV-2 surface protein [3,4]. Due to rare but serious side effects, AZD1222 is no longer recommended unconditionally by the Standing Vaccination Commission (STIKO) of the Robert Koch-Institute for individuals under 60 years of age living in Germany. Vaccinations that have already started with AZD1222 should be continued with an mRNA vaccine after 12 weeks [7].

Up to now, there have been only few studies in which the antibody response of vaccinees to the newly developed mRNA and vector vaccines is examined with identical tests. This makes it difficult to compare the studies.

Here, we investigated the anti-S- and anti-RBD immunoglobulin G (IgG) development after the first vaccination with BNT162b2, mRNA-1273, or AZD1222 as well as after the follow-up vaccination (data for BNT162b2 and mRNA-1273) including determination of IgG avidity. We compared the results with the humoral immune response after infection with SARS-CoV-2 variants of concern (VOCs). We also aimed to prove the development and level of virus-neutralizing antibodies (VNA) with different methods in vaccinees and convalescents. The IgG antibodies could be detected as early as two to three weeks after the first vaccination, and in some cases VNA appeared already in this very early phase. In particular after the second vaccination, an increase in the virus-neutralizing properties and a maturation of the IgG antibodies were observed. These promising results underline the high potential of all examined vaccines.

## 2. Materials and Methods

In this study, sera from eleven SARS-CoV-2 patients and from 100 vaccinees were examined by SARS-CoV-2 specific antibody tests. In the case of the individuals who were vaccinated with the vector vaccine, the sera were obtained shortly before or one week after vaccination and then at weekly intervals for up to one month after vaccination. For those who received the mRNA vaccines, the aim was to have their blood drawn immediately before the second vaccination and then again 3–4 weeks after the second vaccination. All subjects agreed to the serial blood samplings. Moreover, sera from eleven patients with a recent infection caused by VOCs belonging to the SARS-CoV-2 PANGO [8] lineages B.1.1.7 (*n* = 10) and B.1.351 (*n* = 1) were examined. All infections were diagnosed by help of a laboratory developed triplex RT-PCR as described previously [9]. The VOCs were found in January 2021 by subsequent typing of RT-PCR positive samples using probe-based melting curve assays (TIB Molbiol Syntheselabor GmbH, Berlin, Germany) followed by next-generation full-genome sequencing in several cases. The convalescent sera were taken 28–41 days after RT-PCR based diagnosis. The ethics committee of the medical faculty of the Christian-Albrechts-Universität zu Kiel (Kiel, Germany) approved the study design (D467/20, 16 April 2020; amendment 2 February 2021).

### 2.1. SARS-CoV-2 S-Protein-Specific IgG Immunoassays

All sera were tested with the SERION ELISA agile SARS-COV-2 IgG assay (Institut Virion\Serion GmbH, Würzburg, Germany) on a BEP 2000 Advance System (Siemens Healthcare GmbH, Erlangen, Germany) following the manufacturers recommendations. This enzyme-linked immunosorbent assay (ELISA) uses the whole SARS-CoV-2 S-protein as antigen and was previously demonstrated by us to possess a high sensitivity of 96.2% for detection of IgG after PCR-confirmed SARS-CoV-2 infection as well as an excellent specificity of 100% [10]. Furthermore, all samples were tested in parallel with the Abbott SARS-CoV-2 IgG II Quant assay on the Alinity i system strictly following the manufacturer’s instructions (both Abbott, Wiesbaden, Germany). This chemiluminescent microparticle immunoassay detects IgG directed against the RBD of the S-protein. According to the manufacturer, this assay has a sensitivity of 98.8% (sera taken ≥ 15 days after a positive SARS-CoV-2 PCR) and a specificity of 99.6%. Results of the anti-S IgG assay and of the anti-RBD IgG assay were given in Binding Antibody Units (BAU) per milliliter (BAU/mL) by applying the conversion factors determined by the manufacturers based on the measurement of the first WHO International Standard Anti-SARS-CoV-2 Immunoglobulin (NIBSC-Code 20–136) [11]. For both anti-SARS-CoV-2 IgG tests, it was determined by us that borderline results are to be assessed as positive.

### 2.2. SARS-CoV-2 IgG Immunoblots and Analysis of IgG Avidity

All sera obtained from SARS-CoV-2 patients and a serum obtained from each vaccine recipients ≥ 21 days after vaccination, respectively, were tested with and without avidity reagent in the *recom*Line SARS-CoV-2 IgG assay (Mikrogen GmbH, Neuried, Germany) following the manufacturer’s instructions. This immunoblot contains the recombinant nucleo (N)-protein of the seasonal human coronaviruses HCoVs 229E, NL63, OC43, and HKU1 as well as the N-protein, the RBD and the entire S1 subunit of the SARS-CoV-2 S-protein, which appear as separate antigens on a nitrocellulose-strip. The IgG avidity denotes the strength of binding to the viral epitope and may correlate with seroprotection [12,13,14]. The strength of binding was assessed separately for all three SARS-CoV-2 antigens and assigned to the categories low (=1), intermediate (=2), and high (=3). Correspondingly, if several antigens were detected, the mean value of the categorized IgG avidity was calculated. The Dynablot Plus system was generally used and all blots were automatically evaluated with a BLOTrix reader and the *recom*Scan software (all from Mikrogen). The manufacturer indicates an overall diagnostic sensitivity of 96.3% and a specificity of 98.8%. The high diagnostic value of this test was also shown in our previous study [10].

### 2.3. Measurement of SARS-CoV-2-Neutralizing Antibodies

The SARS-CoV-2 convalescent sera and the vaccine recipient sera, which were already characterized in the immunoblot, were also examined for their virus-neutralizing capacities. For this, the cPass™ SARS-CoV-2 Neutralization Antibody Detection Kit (Genescript USA, Inc., Piscataway, NJ, USA) was applied as recommended by the supplier using the BEP 2000 system. This ELISA represents a surrogate virus neutralization test (sVNT, designated here as sVNT #1). In the presence of RBD-specific antibodies, this part of the viral S-protein, which has been conjugated to peroxidase, can no longer bind to the angiotensin-converting enzyme 2 that is attached to the solid phase. After appropriate washing steps, the color reaction turns out to be significantly weaker compared to RBD-antibody-free samples. A signal inhibition ≥ 30% was valued as indicative for the presence of SARS-CoV-2-neutralizing antibodies. Previously, sVNT #1 was found to have an overall sensitivity of 80.3% and a specificity of 99.2% [15] but may overestimate low-neutralizing antibodies [16]. For comparison, the TECO^®^ SARS-CoV-2 Neutralization Antibody ELISA (TECOmedical AG, Sissach, Switzerland) was used according to the manufacturer’s specifications on a BEP 2000 system. This sVNT#2 works according to the same test principle as the cPassTM assay, but uses a signal inhibition ≥ 20% as cut-off.

All convalescent sera and a selection of sera obtained from vaccinees were tested in a laboratory-developed Vero cell-based neutralization assay (cVNT) under BSL-3 conditions as previously reported [10]. However, for capacity reasons, we adapted this assay to a 96-well microtiter format. One day prior infection, 2.5 × 10^4^ Vero cells (order no. 605372, CLS Cell Lines Service GmbH, Eppelheim, Germany) were seeded per well. Then, sera were heat-inactivated at 56 °C for 30 min and diluted from 1:10 to 1:1280 in cell culture medium consisting of Dulbecco’s modified Eagle’s medium supplemented with 3.7 g/L NaHCO_3_, 4.5 g/L glucose, 2 mM L-glutamine, and 1% (*v*/*v*) of Pen-Strep-Fungi mix containing 10,000 U/mL penicillin, 10 mg/mL streptomycin, and 25 µg/mL amphotericin B (all reagents from Bio&SELL GmbH, Feucht, Germany). Thereafter, 25 µL of each serum dilution were mixed with 25 µL of virus suspension containing 50 plaque-forming units of either an own ‘wildtype’ SARS-CoV-2 strain (isolated in spring 2020) or an own B.1.1.7 strain from January 2021. The mix was incubated for 1 h at 37 °C. Then, cells were washed with phosphate-buffered saline (PBS, Bio&SELL), inoculated with 50 μL of the virus-serum dilutions, and incubated on a shaker for 1 h at room temperature, followed by the addition of 50 µL of cell culture medium supplemented with 20% fetal calf serum (*v*/*v*). After four days of incubation under standard conditions, cells were fixed with 4% (*w*/*v*) paraformaldehyde in PBS and stained with an aqueous solution of 0.1% (*w*/*v*) crystal violet and 20% (*v*/*v*) methanol. All dilution steps were tested in triplicates, and cytopathic effect (CPE) formation was compared with an untreated cell control and a viral control. A serum dilution > 1:10 that prevented CPE formation in at least two of three wells compared with the viral control was considered reactive and likely protective. When an exact titer could not be provided, a geometric mean of the two adjacent titers was calculated.

### 2.4. Data Evaluation and Statistical Calculations

Data were statistically analyzed using GraphPad Prism version 9.1.1 software (GraphPad Software, San Diego, CA, USA). The Kruskal-Wallis test, a nonparametric, unpaired, adjusted test, was used to analyze the development of IgGs with high avidity after infection or vaccination and the emergence of VNA as determined by the two sVNTs. Changes in antibody titers after the first and second vaccination and differences in neutralizing capacity after infection with VOCs, both measured in the cVNT, were examined using the Wilcoxon test, a paired nonparametric test. The dependence of the VNA measured in the cVNT on avidity was analyzed using the Mann-Whitney test, an unpaired nonparametric test, and the calculation of Cohen’s kappa. A significance level α of 0.05 was used for all tests. *p* values of <0.05 to <0.0001 are indicated by one (*) to four (****) asterisks. The agreement of the two sVNTs as well as the correlation between the sVNTs and the cVNT were analyzed by calculating Cohen’s kappa and Spearman correlation coefficients, respectively.

## 3. Results

### 3.1. Composition of the Study Groups

This study includes eleven patients after SARS-CoV-2 VOC infection and 100 individuals after vaccination with SARS-CoV-2 mRNA (*n* = 44) or vector (*n* = 56) vaccines. Four individuals had their first serum sample drawn several days after the second vaccination with BNT162b2. Therefore, a booster effect cannot be ruled out in these four individuals. Accordingly, only the results of the second serum sample were taken into account in these cases. Weekly follow-up samples were available from six individuals in the BNT162b2 group and from almost everyone who received the vector vaccine. Due to the different vaccination schemes [7], we were able to examine the sera of the participants who were vaccinated with the mRNA vaccines also after the second homologous vaccination. However, no second serum was available in one participant from the BNT162b2 group. The individuals who received the vector vaccine are currently waiting for their second (heterologous) vaccination or have recently received it. Therefore we cannot yet make any statements about the development of humoral immunity in this subgroup. The composition of the study groups is listed in detail in Table 1.

### 3.2. Anti-S- and Anti-RBD-Specific IgG Response after SARS-CoV-2 Infection and Vaccination

**Figure 1:**
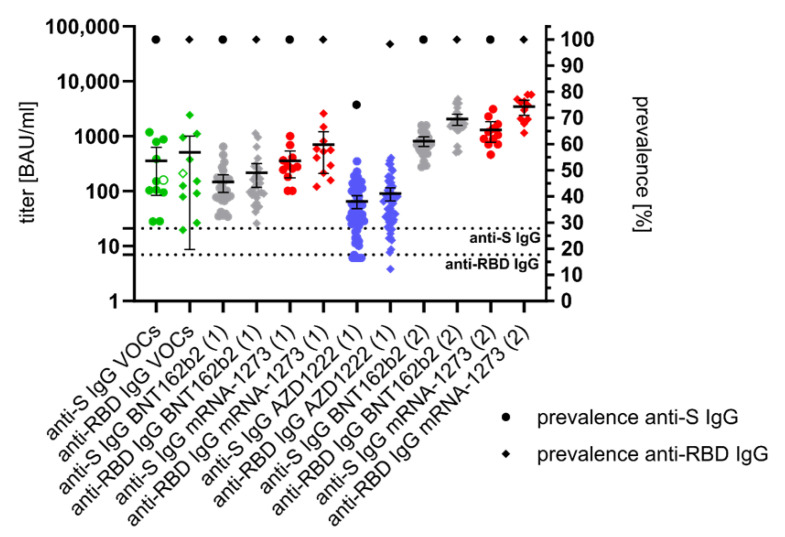
Anti-spike (S)- and anti-receptor-binding domain (RBD) specific immunglobuline G (IgG) response ≥ 21 days after infection with SARS-CoV-2 variants of concern (VOCs: B.1.1.7, filled green dots or diamonds; B.1.351, empty green dots or diamonds) or after first (1) and second vaccination (2) with messenger ribonucleic acid (BNT162b2, grey dots or diamonds; mRNA-1273, red dots or diamonds) and vector (AZD1222, blue dots or diamonds) vaccines. The mean titer and the 95% confidence intervals are given per each study group. All titers were measured in Binding Antibody Units per milliliter (BAU/mL). The prevalence of anti-S/anti-RBD IgG is indicated by a black dot or diamond. The cut-off values for seropositivity (in BAU/mL) of the anti-S and anti-RBD IgG assays are indicated by dotted lines.

Anti-S- and anti-RBD IgG antibodies could be detected in the convalescent sera of all eleven SARS-CoV-2 VOC patients. The mean anti-S IgG titer was 355 BAU/mL and the mean anti-RBD IgG titer was determined with 506 BAU/mL. All individuals who received their first mRNA vaccination had anti-S IgG and anti-RBD IgG, with a mean titer of 147 to 357 BAU/mL and 217 to 706 BAU/mL, respectively. In individuals, who received the vector vaccine, prevalence of anti-S IgG was 75% (mean titer 66 BAU/mL) and of anti-RBD IgG was 98% (mean titer 91 BAU/mL). After the second vaccination, an increase in mean titers of anti-S IgG (BNT162b2: 813 BAU/mL, mRNA-1273: 1305 BAU/mL) and anti-RBD IgG (BNT162b2: 2057 BAU/mL, mRNA-1273: 3472 BAU/mL) was evident (Figure 1).

### 3.3. Kinetics of Anti-S- and Anti-RBD IgG after First SARS-CoV-2 Vaccination

**Figure 2:**
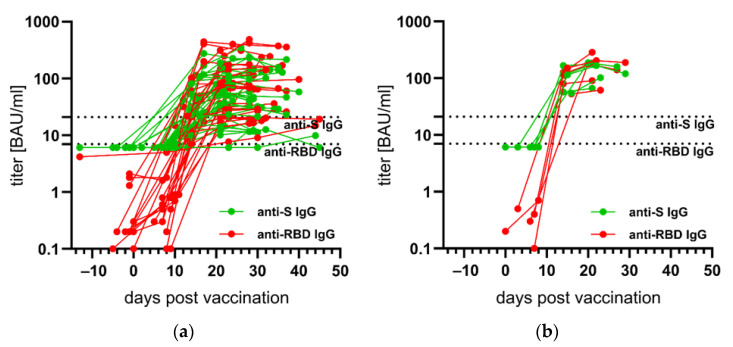
Kinetics of anti-spike (S)- and anti-receptor-binding domain (RBD) immunoglobulin G (IgG) in days before or after the first vaccination with the vector vaccine AZD1222 ((**a**); follow-up samples from 40 vaccinees) and with the messenger ribonucleic acid vaccine BNT162b2 ((**b**); follow-up samples from six vaccinees). All titers were measured in Binding Antibody Units per milliliter (BAU/mL). Connecting lines show the course of the measured values for individual vaccinated persons. The cut-off values for seropositivity (in BAU/mL) of the anti-S- and anti-RBD IgG assays are indicated by dotted lines.

At least two blood samples drawn at short time intervals were available for 40 individuals vaccinated with the vector vaccine AZD1222 and for six individuals vaccinated with BNT162b. The kinetics of anti-S and anti-RBD IgGs were measured accordingly. The first antibodies were already detectable approximately ten days after the immunization. The seronegativity of sera taken shortly before or at the time of vaccination can be interpreted as an indication of the high specificity of both antibody tests. In addition, there is a high probability that those who have been vaccinated were immunologically naïve and had no previous SARS-CoV-2 infection (Figure 2).

### 3.4. Differences in the Anti-N IgG Response between SARS-CoV-2 Infected and Vaccinated Individuals and Development of High Avidity SARS-CoV-2 IgGs after the Second Vaccination

In the *recom*Line immunoassay, anti-N IgG was demonstrated in almost all convalescent sera (91%, 10/11), and anti-RBD as well as anti-S1 IgG was found in 82% (9/11). In the vaccinees, seroreactivity pattern was restricted to anti-S1- and anti-RBD IgG; only one vaccine recipient presented anti-N IgG (Table 2).

**Figure 3:**
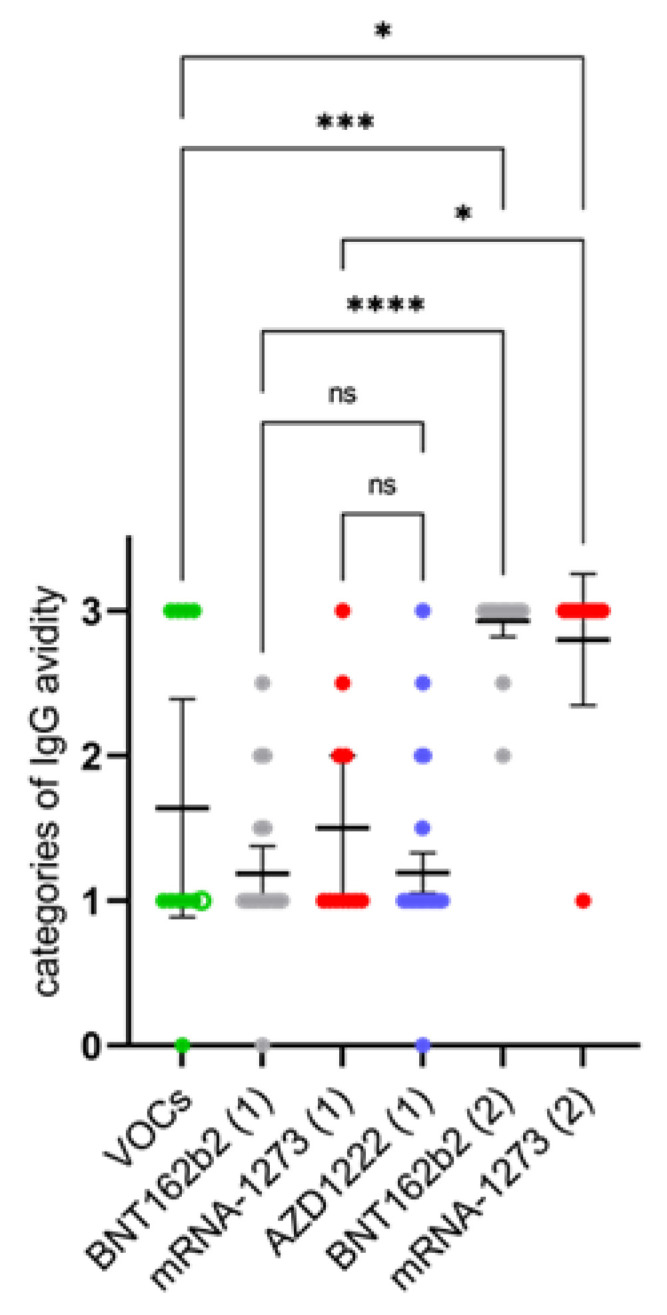
Development of immunoglobulin G (IgG) avidity after infection with SARS-CoV-2 variants of concern (VOCs: B.1.1.7, filled green dots; B.1.351, empty green dot) or after first (1) or second (2) vaccination with messenger ribonucleic acid (BNT162b2; mRNA-1273) or vector vaccines (AZD1222), respectively. The strength of binding was assigned to the categories low (=1), intermediate (=2), and high (=3). Then, the mean value of the categorized IgG avidity was calculated. Ns: not significant; *: *p* < 0.05; ***: *p* < 0.001; ****: *p* < 0.0001. Significance was calculated by using a non-parametric, unpaired, and adjusted test (Kruskal-Wallis test).

The development of SARS-CoV-2 IgG avidity was studied with the *recom*Line assay. Almost all sera taken ≥21 days after the first vaccination showed only low to intermediate IgG avidities (mean indices 1.2 to 1.5; Table 2 and Figure 3). In contrast, IgG of high avidity were found in the sera taken after the second vaccination (mean indices 2.8 to 2.9; Table 2 and Figure 3). Their mean avidity indices were significantly higher than after first vaccination and after infection with VOCs (mean index 1.6; Table 2 and Figure 3).

### 3.5. Appearance of Potent Virus-Neutralizing Antibodies after the Second Vaccination

Surrogate tests should be able to predict the virus-neutralizing properties of a serum. They measure the antibody-mediated prevention of the binding of the viral RBD to the cellular receptor ACE2. The sera of all infected and all vaccinees were examined in the sVNT#1. In this test, almost all VOC patients (*n* = 10; 91%) and all vaccinees after first vaccination with an mRNA vaccine showed relevant inhibition of ACE2 binding (≥30%).

**Figure 4:**
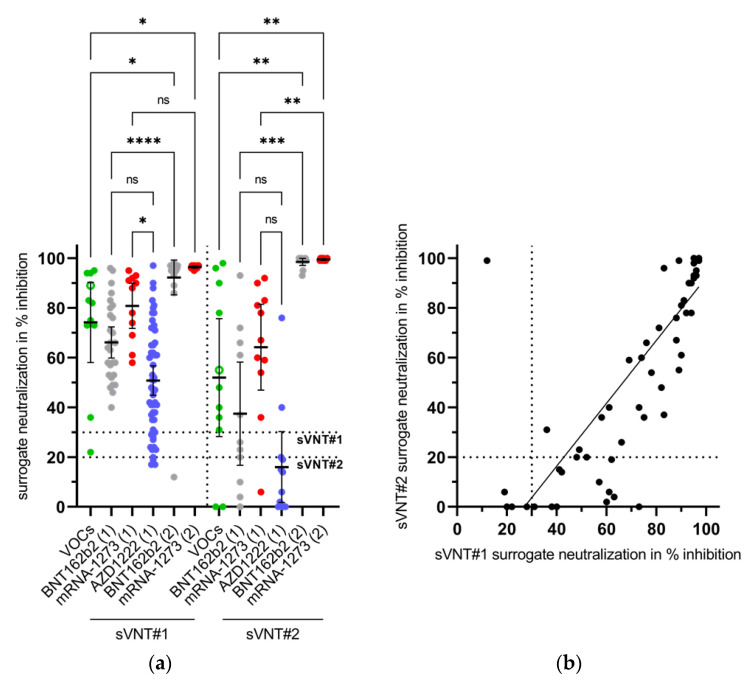
(**a**) Investigation of convalescent sera (after infection with SARS-CoV-2 variants of concern, VOCs) and of vaccinees sera in two surrogate virus neutralization tests (sVNT #1 and #2). These assays measure the antibody-mediated prevention of the binding of the viral receptor-binding domain to the angiotensin-converting enzyme 2. The degree of binding inhibition is given as a percentage and is taken as an indication of the presence of virus-neutralizing antibodies. The mean and the 95% confidence interval are given for each group of patients (VOCs: B.1.1.7 filled green dots; B.1.351 empty green dot) or vaccinees after first (1) or second (2) vaccination with messenger ribonucleic acid (BNT162b2; mRNA-1273) or vector vaccines (AZD1222), respectively. Horizontal dotted lines indicate the cut-offs of both assays. Data were valued for statistical significance by a non-parametric, unpaired, and adjusted test (Kruskal-Wallis test). Ns: not significant; *: *p* < 0.05; **: *p* < 0.01; ***: *p* < 0.001; ****: *p* < 0.0001. (**b**) Quantitative agreement between both sVNTs. The Spearman correlation coefficient was calculated with 0.89.

An initial vaccination with AZD1222 was associated with presence of inhibiting antibodies in 80% of these individuals. For the separate study groups, the mean percentage of the inhibition of binding varies between 50.8% to 96.5% in this sVNT. Sixty-eight sera were also tested with sVNT#2. In this assay, the mean percentage of the inhibition varied from 16 to 99.5% (Figure 4a). The qualitative agreement of both assays was classified as ‘fair’ (Table 3; unweighted Kappa = 0.33), but we found a ‘strong’ correlation of inhibition values in % (Figure 4b; Spearman correlation coefficient = 0.89).

**Figure 5:**
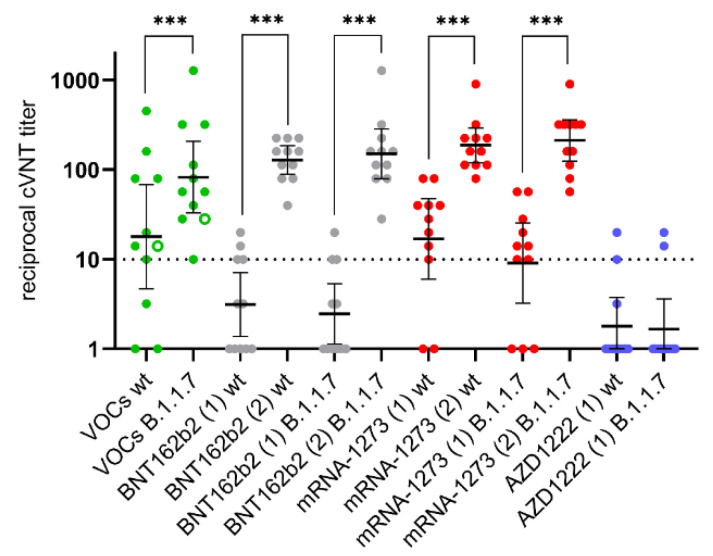
Development of virus neutralizing antibodies against a ‘wildtype’ SARS-CoV-2 strain (wt) and against a B.1.1.7 virus after infection with variants of concern (VOCs: *n* = 10, B.1.1.7, filled green dot; *n* = 1, B.1.351, empty green dot) or after first (1) and second (2) vaccination with with messenger ribonucleic (BNT162b2; mRNA-1273) and vector (AZD1222) vaccines (*n* = 11 age and gender matched vaccinees per vaccine group). Results were obtained in a laboratory-developed Vero-cell based neutralization assay (cVNT) under BSL-3 conditions. A serum dilution of >1:10 is considered as protective. The reciprocal geometric mean titer and the 95% confidence intervals are given. Stars indicate statistical significance (***: *p* < 0.001) calculated in a paired, non-parametric test (Wilcoxon test).

**Figure 6:**
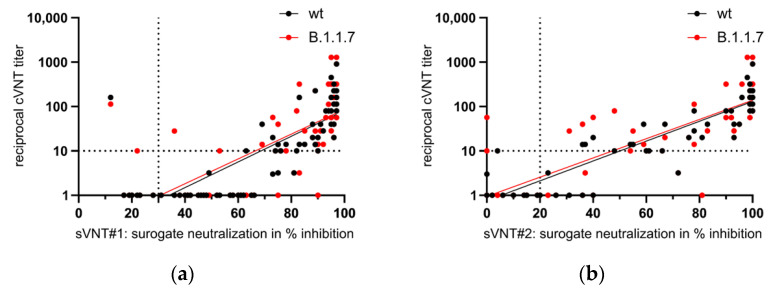
Comparison of the results of the surrogate neutralization tests (sVNT) #1 (**a**) and #2 (**b**) with those of the cell-based neutralization test (cVNT) using a ‘wildtype’ SARS-CoV-2 strain (wt) and a B.1.1.7 strain as antigens. A serum dilution of >1:10 is considered as protective. The decision limits for relevant inhibition of SARS-CoV-2 receptor-binding domain interaction with the human angiotensin-converting enzyme 2 varies between ≥20% (sVNT#2) and ≥30% (sVNT#1). Spearman correlation coefficients were calculated with 0.85 (sVNT#1 vs. cVNT with wt), 0.80 (sVNT#1 vs. cVNT with B.1.1.7), 0.92 (sVNT#2 vs. cVNT with wt), and 0.85 (sVNT#2 vs. cVNT with B.1.1.7), respectively.

**Figure 7:**
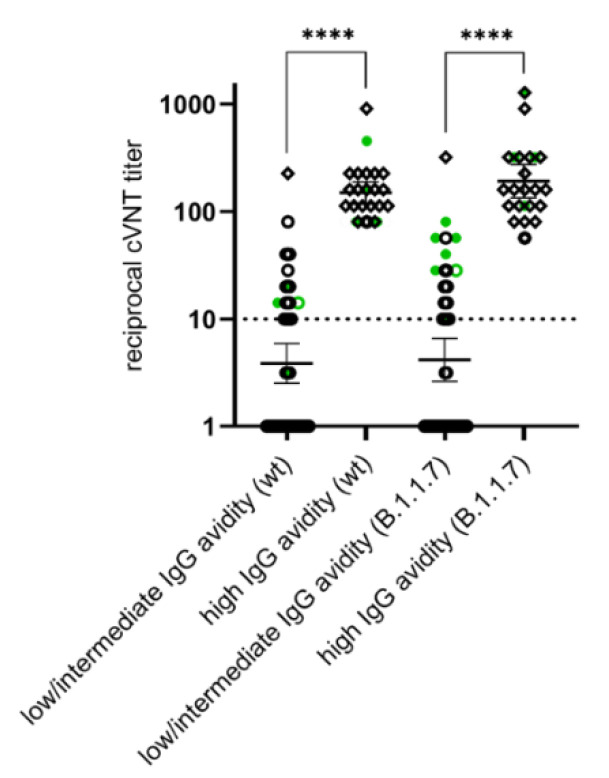
Comparison of immunoglobulin G (IgG) avidity to virus-neutralizing titers obtained with the cell-based neutralization test (cVNT) using a ‘wildtype’ SARS-CoV-2 strain (wt) and a B.1.1.7 virus as antigens. The reciprocal geometric mean titer and the 95% confidence intervals are given. The stars indicate statistical significance (****: *p* < 0.0001) in an unpaired, non-parametric test (Mann-Whitney test). The sera were collected after infection with SARS-CoV-2 variants of concern (VOCs: B.1.1.7, filled green dots; B.1.351, empty green dots) or after first (black empty dots) and second vaccination (black empty diamonds). A dilution of >1:10 is considered as protective.

The eleven convalescent sera were tested for presence of VNA against ‘wildtype’ SARS-CoV-2 and B.1.1.7 in the cVNT. In addition, sera obtained ≥ 21 days after vaccination were included. Since only eleven vaccinees were vaccinated with mRNA-1273, eleven individuals were selected from the other two vaccination groups who roughly corresponded in age and gender to the recipients of mRNA-1273 (Figure 5). Data from additional individual sera that were examined in the cVNT were included in the following test comparisons. Among other things, the results of the cVNT were compared with the results of both sVNTs. This showed that the sVNTs overestimate the neutralizing properties of the sera. Raising the sVNTs cut-off could reduce this overestimation (Figure 6). There was a moderate agreement of qualitative cVNT results and of anti-SARS-CoV-2 IgG avidity categories (‘wildtype’: unweighted Kappa = 0.60; B.1.1.7: unweighted Kappa = 0.58; Table 4). The positive and negative predictive values, which suggest the presence of VNA from a high IgG avidity, were calculated on the basis of the data given in Table 4. They reach 100% and 70%, respectively, regardless of the viral antigen used in the cVNT. In line with this, VNA titer were significantly higher in individuals with highly avid anti-SARS-CoV-2 IgG (Figure 7).

## 4. Discussion

In this study, the humoral immune response after SARS-CoV-2 infection or vaccination was examined. The convalescent sera were obtained from eleven patients after a SARS-CoV-2 infection caused by two VOCs. Even if the identification of the underlying virus lines by sequencing was not carried out in every case, the key mutations detected by RT-PCR and subsequent melting curve analysis indicate that these viruses belong to the SARS-CoV-2 VOC lines B.1.1.7 (*n* = 10) and B.1.351 (*n* = 1). The S-protein of variant B.1.1.7 has seven amino acid (aa) changes, and further three aa have been deleted [17]. The aa change N501Y of the RBD, which is also found in VOC B.1.351, is associated with increased binding to the cellular receptor ACE2 [17]. The S-protein of the B.1.351 variant has seven aa changes, three of them in the RBD, as well as a deletion of three adjacent aa [17]. In this respect, it can be assumed that, in addition to impairing the virus-neutralizing activity of vaccine sera [17], the reliable detection of antibodies in VOC convalescents by S- and RBD-specific tests could also be problematic. In the present study, anti-S- and RBD-specific IgG antibodies were found in all convalescents, the mean titer of which were even higher than after a primary vaccination with BNT162b2 and AZD1222. The results suggest that these assays can reliably monitor humoral immune response after a B.1.1.7 or B.1.351 infection. In almost all cases the vaccination led to the development of IgG against the RBD. However, about 25% of individuals vaccinated with AZD1222 tested negative in the anti-S IgG test. The anti-SARS-CoV-2 specific IgG antibodies were already detectable approximately two weeks after administration of the first vaccination. Thus, the generally high level of seroreactivity and its rapid development indicate a high immunogenicity of the three vaccines. Our results are consistent with a recent publication on IgG responses to various S-protein subunits three weeks after single dose vaccination with the mRNA vaccine BNT162b2 [18]. The slightly lower IgG titers seen from day 21 after the first administration of the vector vaccine in comparison to the mRNA vaccines were also reported in a recent preprint [19,20]. More important than the observed high seroreactivity is the substantial reduction in SARS-CoV-2 infections and infection-related hospital admissions previously reported after a single vaccination with BNT162b2 or AZD1222 [19,21,22,23].

The IgG response of convalescent and vaccinated individuals differs markedly, since post infection, in addition to the anti-S/-RBD IgG antibodies, anti-N IgG can also be detected. This difference is not surprising, but an effect of the vaccination, which solely leads to the expression of the S-protein, against which specific antibodies are then formed. The peculiarity can be used for diagnostic purposes, for example to verify the suspicion of a previous infection or a vaccination breakthrough.

A few weeks after the booster vaccination, the mean IgG titer were not only higher but there was also a marked increase in mean IgG avidity. In contrast, the mean IgG avidities after first vaccination or infection were only low to intermediate. In our previous study, less than 30% of outpatients developed highly avid IgG antibodies several weeks to months after SARS-CoV-2 infection [10]. A high IgG avidity results from a natural maturation process that takes some time and is an indicator of the strength of the interaction between the antibody and its epitope [12,13,14,24]. Due to the short interval between the first vaccination and the booster which is recommended for mRNA vaccines [7], it cannot be ruled out that one dose alone is sufficient for the formation of highly avid IgG after several weeks and prior booster vaccination speeds up the process. Regardless of the causative mechanism, we consider the detection of highly avid anti-S1/-RBD IgG to be a very encouraging sign and an expression of the development of a more robust humoral immunity.

The sera from almost all convalescents and 80 to 100% of the sera from those who were first vaccinated showed an inhibition of RBD-binding to ACE2 of at least 30% in the sVNT#1. These individuals are believed to possess virus-neutralizing antibodies. However, the percentage of inhibition varied. The lowest values were found after a single vaccination with the vector vaccine, the highest values after the second vaccination with the mRNA vaccines. The qualitative agreement and quantitative correlation of the inhibitions measured in both sVNTs was ‘fair’ and ‘strong’, respectively.

In the cVNT, VNA against a canonical non-B.1.1.7 strain (designated here as ‘wildtype’) and against a member of the B.1.1.7 lineage were found in 64% and 91% of the convalescent sera. In this group, the mean neutralizing titer against B.1.1.7 was significantly higher than the mean neutralizing titer against the ‘wildtype’ strain. This can be interpreted as a serological indication of the previous infection with B.1.1.7, which was diagnosed in almost all patients. Such an effect could not be observed for the three groups of vaccinees (statistical data not shown). After the first vaccination, a broader occurrence of VNA against ‘wildtype’ virus and B.1.1.7 was detectable only in one group of mRNA vaccinees, whereas the second vaccination resulted in a significant increase of VNA in cVNT, as also reported by others [3]. A lower in-vitro vaccine-induced virus-neutralizing activity against the B.1.1.7 variant was previously demonstrated for AZD1222 vaccinees while the clinical vaccine efficacy was similar against a ‘wildtype’ strain and the B.1.1.7 variant [25]. In the present study, VNA against the ‘wildtype’ virus and against B.1.1.7 increased to similarly high levels after second vaccination. Our laboratory-developed cVNT [10] is based on the visual assessment of the prevention of the virus-induced CPE by the serum dilution. Thus, this assay evaluates less the reduction in the number of plaques but rather their general prevention and is therefore to be assessed as very conservative. This could explain the poor correlation of lower titers with the sVNT. Data on the diagnostic performance of the sVNTs for detection of low neutralizing sera are inconsistent: both underestimates and overestimates have been reported [15,16,26]. Our results support the observation that the sVNTs overestimate low neutralizing sera. Thus, it might be useful to increase their cut-off values, perhaps even higher than the 50% previously suggested for sVNT#1 [16]. From our point of view, this increase in the sVNT cut-offs would improve their diagnostic accuracy. Then these assays could show their advantages over the cVNT, which consist in the fact that they can be automated, standardized and carried out under routine laboratory conditions.

We found a moderate qualitative agreement between the results of cVNT and the determination of anti-SARS-CoV-2 IgG avidity. Interestingly, individuals with a high VNA had also anti-SARS-CoV-2 IgG of high avidity. Both high-quality antibody properties were detectable after the booster vaccination. It can therefore be assumed that the detection of highly avid anti-SARS-CoV-2 IgG is a good predictor for the presence of virus neutralization capacities in the serum. However, statements on the approximate VNA titer level cannot be derived from the avidity. The association between the presence of highly avid anti-SARS-CoV-2 IgG and VNA should be verified in further, more extensive studies.

A recent investigation from Israel shows that after a complete vaccination with two BNT162b2 doses, the rate of asymptomatic infections is reduced by more than 90% and of symptomatic infections including hospital admissions and deaths by more than 95%. This is particularly noteworthy since almost all infections in Israel are now caused by VOC B.1.1.7 [27]. A more than 95% effectiveness of the BNT162b2 vaccination against severe, critical, or fatal infection with VOCs B.1.1.7 and B.1.351 has been also reported from Qatar [28].

In summary, the results of our study show that the administration of a single vaccine dose already leads to a high rate of anti-S-/-RBD-specific IgG antibodies and therefore a certain protection against (severe) infections can be assumed. However, in order to achieve a more robust immunity, a second vaccination is required.

## Figures and Tables

**Table 1:** Composition of the study groups.

Groups	Number of Individuals	Median Age in Years	Age Span in Years	Gender (Female/Male)	Time Span from RT-PCR or the First/Second Vaccination to Serum Sampling in Days
**SARS-CoV-2 VOC infection**	11	52	18 to 69	6/5	28 to 41
**SARS-CoV-2 vaccination**	100	41	18 to 62	77/23	−13 to 65/15 to 49
mRNA vaccines					
Pfizer/BioNTech (BNT162b2)	33	45	23 to 62	25/8	0 to 37/15 to 49
Moderna (mRNA-1273)	11	48	33 to 61	7/4	22 to 34/20 to 22
vector vaccine					
AstraZeneca (ChAdOx1 nCoV-19/AZD1222)	56	31	18 to 60	45/11	−13 to 65/ no data available yet

mRNA: messenger ribonucleic acid; RT-PCR: one-step reverse transcription real-time polymerase chain reaction for detection of SARS-CoV-2; VOC: variant of concern.

**Table 2:** Pattern of Immunoglobulin G (IgG) reactivity and avidity in patients after infection with SARS-CoV-2 variants of concern (VOCs) or after first (1) and second (2) immunization with messenger ribonucleic acid (BNT162b2; mRNA-1273) or vector (AZD1222) vaccines.

Groups		Anti-N IgG		Anti-RBD IgG		Anti-S1 IgG		High IgG Avidity (Index ≥ 2.5)	
	***n***	***n***	**%**	***n***	**%**	***n***	**%**	***n***	**%**
VOCs	11	10	90.9	9	81.8	9	81.8	4	36.4
BNT162b2 (1)	27	0	0	23	85.2	26	96.3	2	7.4
BNT162b2 (2)	21	0	0	21	100	21	100	20	95.2
m-1273 (1)	11	0	0	11	100	11	100	2	18.2
m-1273 (2)	10	0	0	10	100	10	100	9	90
AZD1222 (1)	56	1	1.8	50	89.3	54	96.4	3	5.4

Results were obtained with the *recom*Line immunoassay. Please note, that the number in the groups differs from the number of the individuals included in the study: If a high IgG avidity was observed after a single vaccination, the serum was not examined again after the second vaccination. Likewise, sera from several individuals were only available after the first/second vaccination. N: nucleoprotein. RBD: receptor-binding domain of the spike (S)-protein. S1: S1 subunit of the S-protein.

**Table 3:** Qualitative agreement of results obtained with both surrogate virus neutralization tests (sVNT).

		sVNT#2 (Decision Limit ≥ 20%)			
		No evidence for VNA	Evidence for VNA	*n*	%
**sVNT#1** **(decision limit ≥ 30%)**	No evidence for VNA	4	1	5	7.4
	Evidence for VNA	11	52	63	92.6
	*n*	15	53	68	
	%	22.1	77.9		

VNA: virus-neutralizing antibodies.

**Table 4:** Qualitative agreement of results obtained in the cell-based virus neutralization test and of IgG avidities measured in the *recom*Line assay.

		IgG Avidity		*n*	%
		Low/Intermediate	High		
**cVNT (wt)**	No evidence for VNA	37	0	37	47.4
	Evidence for VNA	16	25	41	52.6
	*n*	53	25	78	
	%	67.9	32.1		
**cVNT (B.1.1.7)**	No evidence for VNA	36	0	36	46.2
	Evidence for VNA	17	25	42	53.8
	*n*	53	25	78	
	%	67.9	32.1		

The cell-based virus neutralization test (cVNT) was conducted with a SARS-CoV-2 ‘wildtype’ (wt) strain and with a member of the B.1.1.7 lineage as separate antigens. A titer > 1:10 was taken as evidence for presence of virus neutralizing antibodies (VNA).

## Data Availability

The data presented in this study are available on request from the corresponding author.
